# Medical student medium-term skill retention following cardiac point-of-care ultrasound training based on the American Society of Echocardiography curriculum framework

**DOI:** 10.1186/s12947-022-00296-z

**Published:** 2022-10-12

**Authors:** Satoshi Jujo, Brandan I. Sakka, Jannet J. Lee-Jayaram, Akihisa Kataoka, Masaki Izumo, Kenya Kusunose, Atsushi Nakahira, Sayaka Oikawa, Yuki Kataoka, Benjamin W. Berg

**Affiliations:** 1 SimTiki Simulation Center, John A. Burns School of Medicine, University of Hawaii at Manoa, 651 Ilalo St, MEB 212, Honolulu, HI 96813 USA; 2 Department of Anesthesiology, Kameda Medical Center, Chiba, Japan; 3 Division of Cardiology, Department of Internal Medicine, Teikyo University, Tokyo, Japan; 4 Division of Cardiology, Department of Internal Medicine, St. Marianna University School of Medicine, Kawasaki, Kanagawa Japan; 5 Department of Cardiovascular Medicine, Tokushima University Hospital, Tokushima, Japan; 6 Division of Critical Care Medicine, Nara Prefecture General Medical Center, Nara, Japan; 7 Center for Medical Education and Career Development, Fukushima Medical University, Fukushima, Japan; 8 Department of Internal Medicine, Kyoto Min-Iren Asukai Hospital, Kyoto, Japan; 9 Systematic Review Workshop Peer Support Group (SRWS-PSG), Osaka, Japan; 10 Section of Clinical Epidemiology, Department of Community Medicine, Kyoto University Graduate School of Medicine, Kyoto, Japan; 11 Department of Healthcare Epidemiology, Kyoto University Graduate School of Medicine / School of Public Health, Kyoto, Japan

**Keywords:** Point-of-care ultrasound, Handheld ultrasound, Medical education, Medical student, Skill retention

## Abstract

**Background:**

No studies have demonstrated medium- or long-term skill retention of cardiac point-of-care ultrasound (POCUS) curriculum for medical student. Based on the American Society of Echocardiography (ASE) curriculum framework, we developed a blended-learning cardiac POCUS curriculum with competency evaluation. The objective of this study was to investigate the curriculum impact on image acquisition skill retention 8 weeks after initial training.

**Methods:**

This study was a prospective, pre-post education intervention study for first- and second-year medical students, with blinded outcome assessment. The curriculum included a pre-training ASE online module and healthy volunteer hands-on training to obtain 5 views: parasternal long-axis (PLAX), parasternal short-axis (PSAX), apical 4-chamber (A4C), subcostal 4-chamber (S4C), and subcostal inferior vena cava (SIVC) views. Students took 5-view image acquisition skill tests at pre-, immediate post-, and 8-week post-training, using a healthy volunteer. Three blinded assessors rated the image quality using a validated 10-point maximum scoring system. Students used a hand-held ultrasound probe (Butterfly iQ).

**Results:**

Fifty-four students completed hands-on training, and pre- and immediate post-training skill tests. Twenty-seven students completed 8-week post-training skill tests. Skill test score improvement between pre- and 8-week post-training was 2.11 points (95% CI, 1.22–3.00; effect size, 1.13).

**Conclusion:**

The cardiac POCUS curriculum demonstrated medium-term skill retention. The curriculum was sufficient for S4C and SIVC skill retention, but inadequate for PLAX, PSAX, and A4C. Therefore, instructional design modifications or re-training for PLAX, PSAX, and A4C are needed to make the curriculum more effective for clinically relevant skill retention.

**Supplementary Information:**

The online version contains supplementary material available at 10.1186/s12947-022-00296-z.

## Introduction

Cardiac point-of-care ultrasound (POCUS) is a rapid, bedside cardiac ultrasound examination that assesses important cardiovascular pathology. It is increasingly used in clinical practice by multiple specialties including internal medicine, emergency medicine, critical care medicine, and anesthesiology [1–5]. The development of affordable handheld ultrasound (HHU) devices that operate with smartphones or tablets has further increased the utilization of cardiac POCUS [5, 6]. Cardiac POCUS is becoming an essential skill for medical students to learn in preparation for their future clinical practice [7]. Stethoscopes for medical students could be replaced with HHU or “ultrasound stethoscopes” in the foreseeable future to learn cardiovascular clinical examinations [8].

A review on cardiac POCUS education in medical schools included studies from 12 medical schools in the United States and 6 other countries and demonstrated benefits of cardiac POCUS curricular integration [7]. However, instructional designs of reported curricula are highly variable, lacking standardized methodology or competency evaluation with validity evidence [7]. Moreover, competency evaluation in these studies focused on very short-term skill and/or knowledge retention immediately after an initial training, but provided no insights into medium- or long-term retention [9–19]. Developing a curriculum that places greater emphasis on the longevity and durability of a learned skill, rather than immediate recollection or improvement, is important for achieving efficient student learning and effective use of limited instructor time.

To address these issues, the American Society of Echocardiography (ASE) proposed a cardiac POCUS teaching framework for medical students [7]. The ASE framework includes a pre-training didactic education with e-learning (https://aselearninghub.org/), hands-on training, and a competency evaluation. The training goals include enhancing cardiac physical examination skills and augmenting learning of normal anatomy, rather than learning advanced pathology. In our pilot study with 6 pre-clinical medical students, the cardiac POCUS curriculum with the ASE framework demonstrated that students improved image acquisition skills immediately after training, and improved skills were retained 8 weeks after training with a large effect size (ES) [20]. The pilot study confirmed curriculum feasibility and provided rationale for conducting a full-scale study to statistically confirm the skill retention.

The objective of this study was to elucidate learning effects of the curriculum on medium-term skill retention in pre-clinical medical students for future curriculum utilization. We hypothesized that pre-clinical medical students would retain improved cardiac POCUS image acquisition skills 8 weeks after initial training.

## Methods

### Design

This was a prospective, single-group, pre-post educational intervention study with blinded outcome assessment.

### Participants and setting

First- and second-year medical students who had completed the 12-week pre-clinical cardiovascular and pulmonary core curriculum at John A. Burns School of Medicine (JABSOM), University of Hawaii, USA were eligible for the study. We recruited participants through e-mail and public postings. This study was conducted at the JABSOM SimTiki Simulation Center (SimTiki) between September 2019 and June 2020. We continued to recruit participants for the study period even after the statistical sample size was achieved to increase representativeness of the sample. Consequently, 54 participants were included in this study. The University of Hawaii Human Studies Program approved the study (Protocol number: 2019–00265). All participants provided informed consent, and all data were de-identified after collection. No incentives or reimbursements were provided to participants. We carried out this study in accordance with The Code of Ethics of the World Medical Association (Declaration of Helsinki).

### Cardiac POCUS curriculum

**Fig. 1:**
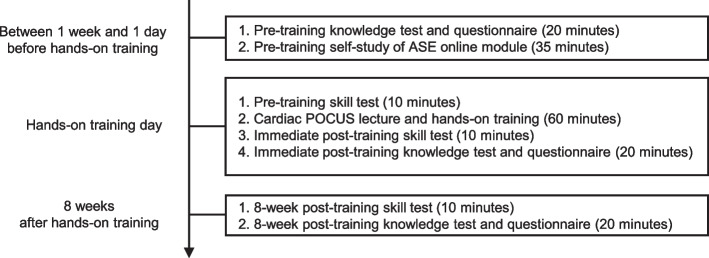
Cardiac point-of-care ultrasound curriculum timeline. *ASE*, American Society of Echocardiography; *POCUS*, point-of-care ultrasound

In our previous pilot study, we developed a basic cardiac POCUS curriculum for pre-clinical medical students based on the ASE-recommended framework that encourages the use of a flipped classroom/blended-learning model with online modules [20]. Student goals for this curriculum were to independently obtain basic cardiac POCUS views in a healthy volunteer and to identify normal anatomic structures seen in cardiac POCUS views. Concepts of curriculum design were underpinned by educational principles for effective learning and skill retention, which include concurrent feedback, deliberate practice, mastery learning, and range of difficulty [21]. Curriculum developers were echocardiography subject matter experts including a Fellow of the European Society of Cardiology (KA), a Fellow of the American Society of Echocardiography (KK), and experienced simulation curriculum developers (BWB and JJL). The curriculum timeline is shown in Fig. 1. The cardiac POCUS curriculum included a pre-training self-study of the ASE cardiac POCUS online module and a hands-on training session with a healthy volunteer. The students used an HHU probe (Butterfly iQ; Butterfly Network, Inc., Guilford, CT, USA) with a 9.7-in. tablet display during the training. Student image acquisition skill and anatomical knowledge were assessed before, immediately after, and 8 weeks after training.

#### ASE cardiac POCUS online module for medical students

The ASE POCUS task force has a free cardiac POCUS online module for medical students (https://aselearninghub.org/). We utilized the ASE online module titled “Cardiovascular Point-of-Care Imaging for the Medical Student and Novice User” as the pre-training didactic. The complete ASE online module comprised 8 sub-modules: Introduction, Basic Anatomy Correlating to Cardiac POCUS Views (module A), Complete Cardiac POCUS Scan (module B), Integrated Cardiac Point-of-Care and Physical Exam (module C), Pathology-I (module D), Pathology-II (module D), Teaching the Teacher (module E), and Standards and Testing (module F). Our pre-training self-study curriculum included the first 4 ASE modules on normal anatomy and physiology (Introduction, modules A, B, and C), which were matched to the learner level of pre-clinical medical students without extensive prior knowledge of cardiac pathology. The 4 ASE modules were designed to be completed in approximately 35 min. Students independently reviewed the online modules 1 day to 1 week before hands-on training.

#### 5 cardiac POCUS views selection

We selected 5 cardiac POCUS views for hands-on training: parasternal long-axis (PLAX), papillary muscle level of parasternal short-axis (PSAX), apical 4-chamber (A4C), subcostal 4-chamber (S4C), and subcostal inferior vena cava (SIVC) views. The 5-view selection was based on recommendations by the World Interactive Network Focused on Critical Ultrasound [2], European Association of Cardiovascular Imaging [22], and ASE [5].

#### Cardiac POCUS hands-on training session

One instructor (SJ) delivered a 30-min interactive 1-on-1 lecture using PowerPoint slides of the ASE online module and a life-size model heart (Cardiac POCUS lecture). Content of the lecture is in Additional file 1, and a pre-recorded video of 5-view image acquisition instruction in the lecture is in Additional file 2 (https://youtu.be/3PfRzsYjKQg) (The video is a short edited version of the actual video for this article.). Following lecture, students engaged in a supervised, 1-on-1 hands-on training of the 5-view image acquisition on a thin, healthy male volunteer for 30 min (Cardiac POCUS hands-on training). The instructor assumed the role of the healthy volunteer during the hands-on training while providing concurrent, verbal, and tactile feedback to guide student skill development. During hands-on training, students deliberately practiced until they obtained each image with clinically acceptable quality. Image acquisition instruction was designed with reference to an imaging protocol in the ASE comprehensive transthoracic echocardiography guidelines and a point-of-care ultrasound textbook [23, 24]. The main instruction points for the 5-view image acquisition are presented in Additional file 3.

### Skill test scoring system

#### Skill test

We assessed image acquisition skill at pre-, immediate post-, and 8-week post-training, using a 10-point maximum skill test scoring system. The skill test is demonstrated in Additional file 4 (https://youtu.be/9KOO_vdNf-c) (One of authors, JJL, played the role of a student in the video). During the skill test, students demonstrated the 5 cardiac POCUS views on the same single healthy volunteer as in the hands-on training without guidance. Students were given 2 min to obtain each view, for a total of 10 min for 5 views. Once the students found their “best” view, they pressed the record button on the tablet for a 5-second clip. Students were allowed to record a maximum of 2 clips for each view. If they had 2 recordings, they selected a single recording for evaluation. We utilized the Butterfly iQ application predefined cardiac ultrasound preset for gain and other ultrasound imaging parameters [25]. We preset the imaging depth to 16 cm for PLAX and PSAX, 18 cm for A4C, and 20 cm for S4C and SIVC. The healthy volunteer was in the left decubitus position for PLAX, PSAX, and A4C, and the supine position with bent knees for S4C and SIVC. The healthy volunteer controlled his respiratory rate at 6 per min and held his breath for 5 seconds when the view recording started.

#### 10-point maximum skill test scoring system

**Fig. 2:**
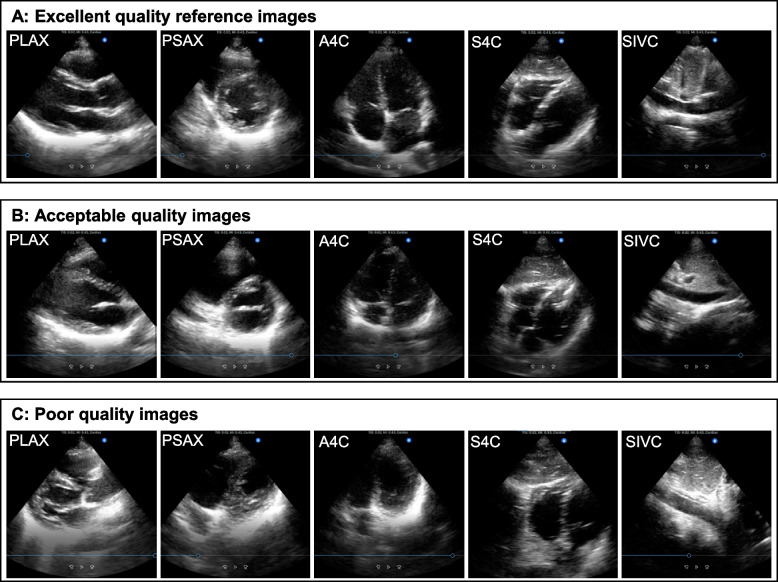
Excellent quality reference (**A**) and examples of acceptable (**B**) and poor quality (**C**) images of 5 cardiac POCUS views. Excellent quality reference images (**A**) refer to 5 cardiac POCUS views obtained by the cardiologist (MI) on the healthy volunteer (SJ) used for all skill tests. Examples of acceptable (**B**) and poor quality (**C**) images refer to the 5 views obtained by medical students on the healthy volunteer. Adapted from Jujo et al. [20]. *A4C*, apical 4-chamber view; *PLAX*, parasternal long-axis view; *POCUS*, point-of-care ultrasound; *PSAX*, papillary muscle level of parasternal short-axis view; *SIVC*, subcostal inferior vena cava view; *S4C*, subcostal 4-chamber view

We developed a 10-point maximum scoring system by modifying an existing assessment tool for transthoracic echocardiography views in our previous pilot study [20, 26]. The scoring system was designed to assess the 5-view image quality for rapid bedside cardiac assessment, not for a formal diagnostic comprehensive echocardiography examination. The 10-point maximum skill test scoring system rated the 5 views; each received a score ranging from 0 to 2 points (Table 1). Each view was assessed as excellent (2 points), acceptable (1 point), or poor (0 point) for cardiac POCUS use. The scores from 5 views were summed for a 10-point maximum test score. Excellent quality reference images and videos of the 5 views obtained by a cardiologist (MI) on the healthy volunteer are in Fig. 2A and Additional file 5 (https://youtu.be/DrPp2C7ET8c). Examples of acceptable and poor quality images and videos obtained by participants are in Fig. 2B, C, and Additional files 6 and 7 (https://youtu.be/fCuYUNW87XY, https://youtu.be/25wj2ml51Pk), respectively. After de-identifying skill test clips including information of pre-, immediate post-, and 8-week post-training, we downloaded the de-identified clips in an electronic database and arranged the clips in randomized order using the random number table in Microsoft Excel for blinded assessment. Three independent blinded raters scored the image quality using the scoring system, and the average of the scores from the 3 raters was then utilized as a representative score. The 3 raters were echocardiography experts. In our pilot study, the skill test scoring system demonstrated excellent interrater reliability and test-retest reliability of the 3 raters [20]. It also demonstrated outstanding discriminatory ability between novices and experts for echocardiography in a validation study using skill test scores from 60 medical students in our pilot study and the current study (Additional file 8).

**Table 1:** 10-point maximum skill test scoring system

5 cardiac POCUS views	Points	Image quality criteria	
PLAX	2	Excellent:	All 7 chambers and anatomical structures (LA, LV, LVOT, RV, AV, MV, and IVS) visualized or similar to the excellent quality reference^a^.
	1	Acceptable:	One chamber (LA, LV, or RV) severely foreshortened or 1 anatomical structure (LVOT, AV, MV, or IVS) not visualized well.
	0	Poor:	Any 2 chambers or structures (LA, LV, LVOT, RV, AV, MV, and IVS) severely foreshortened/not visualized well, the left and right sides of the image are flipped, raters do not recognize the view as a parasternal long-axis view, or no image obtained.
PSAX	2	Excellent:	All 4 chambers and anatomical structures (round LV, RV, papillary muscles, and IVS) visualized or similar to the excellent quality reference^a^.
	1	Acceptable:	One chamber or anatomical structure (round LV, RV, papillary muscles, or IVS) not visualized well, oval LV, significant lateral wall drop out of LV compared with the excellent quality reference^a^, or mitral level of parasternal short-axis view.
	0	Poor:	Any 2 chambers or anatomical structures (round LV, RV, papillary muscles, and IVS) not visualized well, apical level or aortic valve level of parasternal short-axis view, the left and right sides of the image are flipped, raters do not recognize the view as a parasternal short-axis view, or no image obtained.
A4C	2	Excellent:	All 8 chambers and anatomical structures (LA, LV, RA, RV, MV, TV, IAS, and IVS) visualized or similar to the excellent quality reference^a^.
	1	Acceptable:	One chamber (LA, LV, RA, or RV) severely foreshortened, 1 anatomical structure (MV, TV, IAS, or IVS) not visualized well, aortic outflow added (5-chamber view), or significant lateral wall drop out of LV compared with the excellent quality reference^a^.
	0	Poor:	Any 2 chambers or anatomical structures (LA, LV, RA, RV, MV, TV, IAS, and IVS) not visualized well, the left and right sides of the image are flipped, raters do not recognize the view as an apical 4-chamber view, or no image obtained.
S4C	2	Excellent:	All 7 chambers and anatomical structures (LA, LV, RA, RV, IAS, IVS, and liver) visualized or similar to the excellent quality reference^a^. The left and right side flipped image does not affect the subcostal 4-chamber view scoring.
	1	Acceptable:	One chamber or anatomical structure (LA, LV, RA, RV, IAS, IVS, or liver) severely foreshortened/not visualized well or aortic outflow added (5-chamber view).
	0	Poor:	Any 2 chambers or anatomical structures (LA, LV, RA, RV, IAS, IVS, and liver) not visualized well, raters do not recognize the view as a subcostal 4-chamber view, or no image obtained.
SIVC	2	Excellent:	IVC visualized in a longitudinal fashion, connection of IVC to RA visualized clearly, and IVC diameter > = 1.0 cm at 2 cm from the RA-IVC junction, or similar to the excellent quality reference^a^. The left and right sides flipped image does not affect the subcostal IVC view scoring.
	1	Acceptable:	IVC diameter > = 1.0 cm at 2 cm from the RA-IVC junction, but no clear connection of IVC to RA, or IVC not visualized in a longitudinal fashion.
	0	Poor:	IVC diameter < 1.0 cm at 2 cm from the RA-IVC junction, descending aorta imaged instead of IVC, raters do not recognize the view as a subcostal IVC view, or no image obtained.

The 2-point maximum scores for each of the 5 cardiac POCUS views are added for the 10-point maximum skill test score

*AV* aortic valve, *A4C* apical 4-chamber view, *IAS* interatrial septum, *IVC* inferior vena cava, *IVS* interventricular septum, *LA* left atrium, *LV* left ventricle, *LVOT* left ventricle outflow tract, *MV* mitral valve, *PLAX* parasternal long-axis view, *POCUS* point-of-care ultrasound, *PSAX* papillary muscle level of parasternal short-axis view, *RA* right atrium, *RV* right ventricle, *SIVC* subcostal inferior vena cava view, *S4C* subcostal 4-chamber view, *TV* tricuspid valve

^a^Excellent quality reference refers to an image obtained by the cardiologist (MI) on the healthy volunteer used for all skill tests (Fig. 2A and Additional file 5). Adapted from Jujo et al. [20]

### Knowledge test scoring system

We assessed the anatomical knowledge of students before, immediately after, and 8 weeks after training, using an identical knowledge test on Google Forms (Fig. 1). The knowledge test consisted of 40 multiple-choice questions identifying normal anatomic structures seen in the 5 cardiac POCUS views. The 40-point maximum knowledge test scoring system is in Additional file 9. This scoring system demonstrated outstanding discriminatory ability between novices and experts for echocardiography in a validation study using knowledge test scores from 59 medical students in our pilot study and the current study (Additional file 10).

### Outcome measures

We measured the following curriculum learning effect outcomes: The primary outcome was [i] and secondary outcomes were [ii]–[viii].

#### Skill test score improvement

[i] skill test score difference between pre-training and 8-week post-training and [ii] the difference between pre-training and immediate post-training.

#### Knowledge test score improvement

[iii] knowledge test score difference between pre-training and 8-week post-training and [iv] the difference between pre-training and immediate post-training.

#### 5-point Likert scale questionnaire

We administered 5-point Likert scale questionnaires using Google Forms to measure [v] overall curriculum satisfaction, [vi] the ASE online module satisfaction, and [vii] hands-on training satisfaction at immediate post- and 8-week post-training. Questionnaires also assessed [viii] student motivation to purchase a personal HHU at pre-, immediate post-, and 8-week post-training.

#### Subgroup analysis

Based on our pilot study findings of individual skill retention variation [20], we planned to perform subgroup analyses to investigate factors that affected skill retention. When we found significant skill test score variation at 8-week post-training by visual inspection, we examined demographic factors between students with a skill test score of 5 or higher and less than 5 at 8-week post-training to investigate the reason for score variation.

### Interrater reliability of the skill test scoring system

We assessed interrater reliability of the skill test scoring system with intraclass correlation coefficient (ICC) using all skill test scores (pre-, immediate post-, and 8-week post-training).

### Sample size and power calculation

Sample size calculation was based on our pilot study with 6 pre-clinical medical students [20]. The pilot study showed that the mean skill test score difference between pre-training and 8-week post-training was 2.28 points [standard deviation (SD), 4.44]. Using this estimate, 25 participants were required to provide 80% power with a one-sided alpha level of 0.05. Assuming an approximately 15% participant withdrawal from the study, the sample size required was 29. Participation in the study was voluntary; thus, sampling was not random. To increase representativeness of the sample and precision of the outcome measures, we continued to recruit participants for the planned study period even after the statistical sample size was achieved.

### Statistical analysis

All statistical analyses were performed using BellCurve for Excel (Social Survey Research Information Co., Ltd.). All numeric variables are presented as mean and SD, or median and interquartile range. Mean difference (MD) between pre- and 8-week post-training data was calculated with an unpaired t-test and presented as mean and SD with 95% confidence interval (CI) and the ES. MD between pre- and immediate post-training data was calculated with a paired t-test and presented as mean and SD with 95% CI and the ES. We interpreted the clinical significance of ES according to Cohen’s ES guidelines (ES of 0.2–0.5 = small ES, 0.5–0.8 = moderate ES, and > 0.8 = large ES) [27, 28]. ICC estimates and their 95% CIs were calculated based on a mean rating (k = 3), absolute agreement, two-way random-effects model for interrater reliability and two-way mixed-effects model for test-retest reliability [29].

This manuscript adheres to the Guideline for Reporting Evidence-based practice Educational interventions and Teaching (GREET) with the GREET checklist (Additional file 11) [30].

## Results

### Early termination of study due to COVID-19 restrictions

In March of 2020, due to the coronavirus disease 2019 (COVID-19) pandemic, the University of Hawaii Human Studies Program recommended pausing research that involved any face-to-face interaction until after the crisis abated for researcher and study participant safety. In response to the recommendation, we terminated our study on March 16, 2020.

### Participant characteristics

**Fig. 3:**
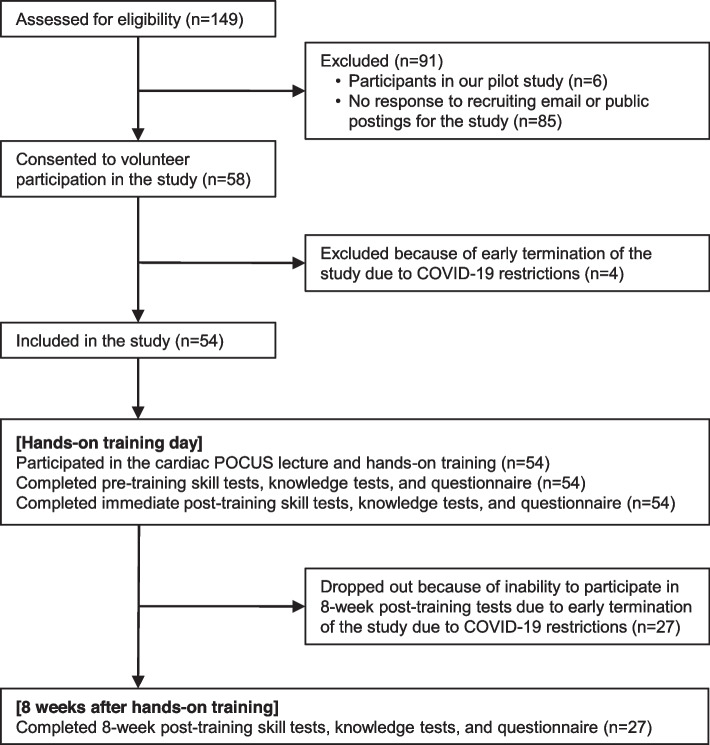
Study flow

Of the 149 eligible students at JABSOM, 54 participated in the study. All 54 students completed the pre-training assessment, hands-on training session, and immediate post-training assessment. Twenty-seven students (50%) completed the 8-week post-training assessment, whereas the remaining 27 (50%) did not because of early study termination (Fig. 3). Student characteristics and cardiac ultrasound training experience are in Table 2. Subgroup characteristics of students with all tests completed (*n* = 27) and without 8-week post-training tests completed (*n* = 27) are also in Table 2. No students received structured ultrasound hands-on training or lecture before study participation.

**Table 2:** Participant characteristics with subgroup characteristics of students with all tests completed and without 8-week post-training tests completed

	Students with all tests completed (*n* = 27)	Students without 8-week post-training tests completed (*n* = 27)	All participating students (*n* = 54)
1st year/2nd year student	17 (63)/10 (37)	10 (37)/17 (63)	27 (50)/27 (50)
Age (years)	25.0 ± 3.6	25.1 ± 2.5	25.1 ± 3.1
Female	10 (37)	17 (63)	27 (50)
Left hand dominant	2 (7)	2 (7)	4 (7)
Pre-training skill test score (10-point maximum)	3.20 ± 1.48	2.21 ± 1.75	2.70 ± 1.68
Pre-training knowledge test score (40-point maximum)	17.1 ± 9.5	13.8 ± 9.6	15.4 ± 9.6
First-choice residency program	IM 6 (22) GS 6 (22) EM 5 (19) Ortho 4 (15) Peds 3 (11) Others 2 (7) Undecided 1 (4)	IM 7 (26) Peds 4 (15) EM 3 (11) GS 3 (11) FM 3 (11) OB/GYN 3 (11) Others 3 (11) Undecided 1 (4)	IM 13 (24) GS 9 (17) EM 8 (15) Peds 7 (13) FM 4 (7) Ortho 4 (7) Others 7 (13) Undecided 2 (4)
Previous ultrasound training experience			
Structured ultrasound hands-on training or lecture	0 (0)	0 (0)	0 (0)
Unstructured ultrasound hands-on training or lecture	15 (56)	8 (30)	23 (43)
Cardiac ultrasound on patients	1 (4)	0 (0)	1 (2)
Observation of a cardiac ultrasound on patients	16 (59)	14 (52)	30 (56)
Cardiac ultrasound on healthy volunteers	21 (78)	13 (48)	34 (63)
Cardiac ultrasound on simulators	2 (7)	1 (4)	3 (6)
Experience using HHU (Butterfly iQ)	4 (15)	4 (15)	8 (15)
Completion of pre-training self-study of the ASE online module	27 (100)	26 (96)	53 (98)
ASE online module review between immediate post-training tests and 8-week post-training tests	6 (22)	NA	NA
Review of textbooks or websites other than the ASE module between immediate post-training tests and 8-week post-training tests	11 (41)	NA	NA
Additional hands-on training between immediate post-training tests and 8-week post-training tests	0 (0)	NA	NA

Data are presented as mean ± SD or *n* (%)

*ASE* American Society of Echocardiography, *EM* emergency medicine, *FM* family medicine, *GS* general surgery, *IM* internal medicine, *IR* interventional radiology, *NA* not applicable, *Ortho* orthopedics, *Peds* pediatrics

### Outcome measures

**Fig. 4:**
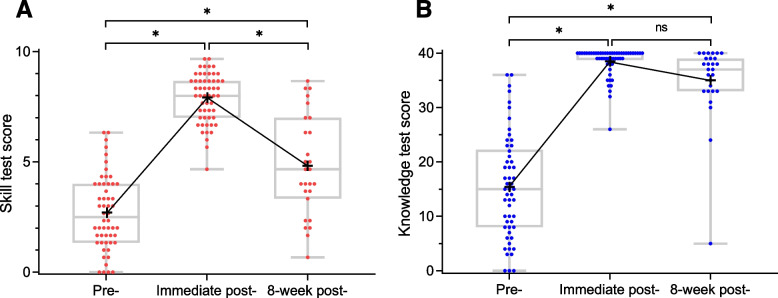
Mean skill (**A**) and knowledge (**B**) test scores with median and individual scores. Red (  ) and blue dots (  ) indicate individual scores. Boxplots indicate minimum, maximum, median, lower, and upper quartiles. Crosses (+) indicate mean. *ns*, not significant. ^⁎^*p* < .0001

Mean skill and knowledge test scores with median and individual scores are shown in Fig. 4. Breakdown of skill test scores for the 5 views are summarized in Table 3. The breakdown scores in student groups with all tests completed (*n* = 27) and without 8-week post-training tests completed (*n* = 27) are in Additional file 12.

**Table 3:** Mean skill test scores and breakdown scores for 5 cardiac POCUS views

	Pre-training	Immediate post-training	8-week post-training
Skill test score^a^	2.70 ± 1.68	7.91 ± 1.12	4.81 ± 2.28
PLAX score^b^	0.51 ± 0.78	1.61 ± 0.57	0.73 ± 0.84
PSAX score^b^	0.49 ± 0.63	1.69 ± 0.39	0.52 ± 0.79
A4C score^b^	0.32 ± 0.47	1.11 ± 0.49	0.68 ± 0.65
S4C score^b^	1.06 ± 0.73	1.54 ± 0.47	1.40 ± 0.61
SIVC score^b^	0.33 ± 0.72	1.97 ± 0.12	1.49 ± 0.83

Data are presented as mean ± SD

The 2-point maximum scores for each of the 5 cardiac POCUS views are added for the 10-point maximum skill test score

*A4C* apical 4-chamber view, *PLAX* parasternal long-axis view, *PSAX* papillary muscle level of parasternal short-axis view, *SIVC* subcostal inferior vena cava view, *S4C* subcostal 4-chamber view

^a^10-point maximum score

^b^2-point maximum score

#### Skill test score improvement

[i] The skill test score difference between pre-training and 8-week post-training was 2.11 points (95% CI, 1.22–3.00; large ES of 1.13), and [ii] the difference between pre-training and immediate post-training was 5.20 points (95% CI, 4.71–5.70; large ES of 3.68).

#### Knowledge test score improvement

[iii] The knowledge test score difference between pre-training and 8-week post-training was 19.6 points (95% CI, 15.4–23.8; large ES of 2.24), and [iv] the difference between pre-training and immediate post-training was 23.1 points (95% CI, 20.5–25.6; large ES of 3.30).

#### Post-training questionnaire

The mean 5-point Likert rating of [v] overall curriculum satisfaction were 4.9 ± 0.6 and 4.8 ± 0.4, [vi] the ASE online module satisfaction were 3.9 ± 0.6 and 4.1 ± 0.6, and [vii] hands-on training satisfaction were 4.9 ± 0.6 and 5.0 ± 0.2 at immediate post- and 8-week post-training, respectively (mean ± SD). The mean 5-point Likert ratings of [viii] student motivation to purchase a personal HHU were 3.4 ± 0.9, 4.0 ± 0.8, and 4.0 ± 0.9 at pre-, immediate post-, and 8-week post-training, respectively (mean ± SD).

#### Subgroup analysis

We found significant skill test score variation at 8-week post-training by visual inspection (Fig. 4A). Subgroup characteristics of students with a skill test score of 5 points or higher and less than 5 points at 8-week post-training are in Additional file 13. Compared to students with less than 5 points, students with 5 points or higher tended to have higher scores on pre-training knowledge tests, include more males, and first-year students. All students in both subgroups received no additional hands-on training between immediate post- and 8-week post-training tests. Because of the small subgroup sample size, we could not reach any conclusions about individual skill retention variation.

### Interrater reliability of the skill test scoring system

Interrater reliability of the skill test scoring system assessed using all 135 score results of 10-point maximum skill tests from the 54 students was excellent (ICC, 0.93; 95% CI, 0.76–0.97).

## Discussion

The ASE-recommended cardiac POCUS curriculum demonstrated medium-term retention, not only short-term, of cardiac POCUS image acquisition skills. This study is the first to provide learning effect evidence on longevity and durability of learned skill from a cardiac POCUS curriculum in a scientifically robust method, including collecting validity evidence for scoring systems. The curriculum was developed with the ASE medical education framework and linked with competency evaluation. It can be utilized as a standardized introductory cardiac POCUS curriculum for medical students or other novices. With reference to our study findings, educators can develop and institute effective and efficient curricula at their schools.

### Previous research

The 8 weeks post-training timeframe for retention assessment in our study was chosen based on 2 previous studies [31, 32]. Fisher et al. studied skill retention after cadaver training for pigtail thoracostomy, femoral line placement, and endotracheal intubation with medical students, and reported that improved skills declined between 6 and 12 weeks [31]. Fisher et al. concluded that a refresher course should be considered when teaching complex technical skills. The study findings were consistent with skill degradation in 3 views (PLAX, PSAX, and A4C) in our study. Rappaport et al. investigated medical student temporal degradation of image acquisition skill 1, 4, and 8 weeks after cardiac, lung, and vascular ultrasound training [32]. Skill decay occurred at 8 weeks for PLAX and at 4 weeks for PSAX and SIVC, whereas lung, and vascular ultrasound skills did not decline statistically. Rappaport et al. assumed that the skill decay for cardiac images was due to the relatively higher complexity of the image acquisition compared with simpler pleural and vascular image acquisition. Interestingly, medical students in Rappaport’s study experienced SIVC skill degradation, which did not occur in our study. A possible reason for the conflicting results is that simultaneous integration of 3 different ultrasound trainings in a single curriculum may have imposed a cognitive load on the novice learners that surpassed their memory capacity [33]. Alternatively, our blended-learning curriculum with the ASE framework may have contributed to the retention difference. Rappaport’s instructional design included a 1-hour lecture and a 1-hour supervised hands-on training without a pre-training self-study. Rappaport did not report concepts of curriculum design, detailed teaching methods, and validity evidence of assessment tools, which were essential to an effective curriculum development for skill retention [21]; these were reported in our study.

### Future echocardiography training in medical school

During the COVID-19 pandemic, medical schools worldwide have been facing unprecedented challenges in education delivery. Medical schools are limiting ward-based teaching and shifting their teaching format from face-to-face to online. To address this rapidly changing educational environment, development of standardized online-based teaching with scientific validation is urgently needed for all medical students who are missing their usual, previously planned education. Several medical students who were scheduled to participate in our cardiac POCUS curriculum missed additional hands-on practice because of early study termination due to COVID-19 restrictions. Future echocardiography training in medical school should be online- and/or simulation-based education that minimizes face-to-face and bedside teaching for both student and patient safety [34]. The ASE recommends 3 core components of cardiac POCUS education; didactic education, hands-on training, and image interpretation [7]. Didactic education and image interpretation teaching can be provided entirely online. With regard to hands-on training, instructor-led face-to-face teaching with real-time feedback is still needed for image acquisition skill development [9, 35]. In our study, we utilized the ASE online module to minimize lecture time and maximize hands-on practice time on the hands-on training day. However, a face-to-face instruction on 5-view image acquisition for 10 min, which could be provided online, was still needed. This is because the ASE online module did not provide a detailed instruction on the item. If the ASE online module included the instruction, our lecture session could be shortened by 10 min. We encourage undergraduate medical education program directors to utilize the ASE module with our video instruction on 5-view image acquisition to develop more comprehensive online-based curricula in the future.

### Limitations

First, the primary outcome may represent attrition bias because 27 participants did not complete the 8-week post-training assessment. However, the dropout was unavoidable due to COVID-19 restrictions, which was unlikely to have induced systematic differences (e.g., selection and volunteer bias) between the 27 participants who followed up at 8 weeks and the 27 did not; the primary outcome result may be considered representative of all 54 participants.

Second, this is a single-center study with small sample size; therefore, subsequent investigations with a larger cohort and in multiple medical schools are needed to validate and generalize our findings [36, 37]. However, this study demonstrated medium-term skill retention with a large ES and the 95% CI not including zero, indicating clinically and statistically significant curriculum effects. Sample size calculations for educational interventions suggest to enroll sample sizes of 25 for large ES of educational interventions; this concept validates our preplanned sample size and results based on actual enrollment as meaningful [20, 38].

Third, breakdown of the skill test scores showed that PLAX, PSAX, and A4C scores at 8-week post-training did not achieve 1.0 point, indicating poor or unacceptable quality for clinical use. One plausible reason for this is the additional difficulty of obtaining those 3 views. Parasternal and apical views (PLAX, PSAX, and A4C) require probe placement in the intercostal space avoiding the lung and ribs with careful probe manipulation. Compared with these views, subcostal views (S4C and SIVC) are obtained by placing the probe under the xiphoid process without fine probe manipulation in the intercostal space. Subcostal views were therefore relatively easy to obtain, required less precise probe manipulation, and may have been easier techniques to recall. Parasternal and apical views were in contrast challenging to obtain and may have been difficult for novice students to recall the technique. To develop more effective curricula for retaining skills, instructional design modifications or refresher training of the parasternal and apical views are needed.

Fourth, this study did not include a control group. Therefore, our curriculum did not demonstrate its superiority over other curricula. When developing the study protocol, we considered designing comparative study with a control group. However, neither a standardized curriculum nor curriculum with medium- or long-term skill retention effects was available for comparison. Thus, we addressed the development of a standardized curriculum that could demonstrate lasting educational benefits using the ASE curriculum framework. Future comparative studies are encouraged to utilize our curriculum as a control group for more effective and practical curriculum development.

Fifth, the skill test scoring system evaluated image acquisition skills to adjust probe position but did not assess image optimization skills to adjust ultrasound imaging parameters, patient position, or controlling patient breathing, because these adjustments were preset by the study protocol. Therefore, whether the students were able to obtain similar quality images to the study results without the study presetting is unknown. When integrating cardiac POCUS curricula into medical schools, workflow training incorporating these adjustments is warranted for curriculum comprehensiveness and practicality.

Sixth, the students in this study were volunteer participants, who may have been highly-motivated students to learn cardiac POCUS. Therefore, student skill retention might in this study be overestimated [39].

Finally, we only investigated medium-term retention 8 weeks after initial training; we did not explore longer term retention after more than 1 year. Future studies that follow up the durability of acquired skills for at least 1 year are warranted to develop a curriculum with long-term skill retention.

## Conclusions

Our cardiac POCUS blended-learning curriculum with the ASE framework demonstrated medium-term retention of image acquisition skills 8 weeks after the initial training with a large ES of skill test score improvements. Educators can utilize the curriculum design as a reference while developing standardized curricula efficient for both trainees and trainers. Breakdown of skill test scores showed that the image quality of S4C and SIVC at 8-week post-training was acceptable for clinical use; however, that of PLAX, PSAX, and A4C was not. Therefore, instructional design modifications of the 3 views or refresher training are needed to make this curriculum more effective for clinically relevant skill retention.

## Supplementary Information

**Additional file 1.** Content of cardiac POCUS lecture.

**Additional file 2.** 5-view image acquisition instruction.

**Additional file 3.** Main instruction points for 5 cardiac POCUS views image acquisition.

**Additional file 4.** The skill test.

**Additional file 5.** Excellent quality reference videos of the 5 cardiac POCUS views obtained by the cardiologist (MI) on the healthy volunteer.

**Additional file 6.** Examples of acceptable quality videos of the 5 cardiac POCUS views obtained by participants.

**Additional file 7.** Examples of poor quality videos of the 5 cardiac POCUS views obtained by participants.

**Additional file 8.** Discriminatory ability of skill test scoring system.

**Additional file 9.** 40-point maximum knowledge test scoring system.

**Additional file 10.** Discriminatory ability of knowledge test scoring system.

**Additional file 11.** GREET checklist.

**Additional file 12.** Mean skill test scores and breakdown scores in student groups with all tests completed and without 8-week post-training tests completed.

**Additional file 13.** Subgroup characteristics of students.

## Data Availability

The datasets used and/or analyzed during the current study are available from the corresponding author on reasonable request.

## Abbreviations

ASE: American Society of Echocardiography
A4C: Apical 4-chamber view
CI: Confidence interval
COVID-19: Coronavirus disease 2019
HHU: Handheld ultrasound
ICC: Intraclass correlation coefficient
JABSOM: John A. Burns School of Medicine
MD: Mean difference
PLAX: Parasternal long-axis view
POCUS: Point-of-care ultrasound
PSAX: Papillary muscle level of parasternal short-axis view
SIVC: Subcostal inferior vena cava view
SD: Standard deviation
S4C: Subcostal 4-chamber view
