# Supplementary material for: Medical student medium-term skill retention following cardiac point-of-care ultrasound training based on the American Society of Echocardiography curriculum framework
Source: Cardiovasc Ultrasound. 2022 Oct 12;20:26. doi: 10.1186/s12947-022-00296-z (PMC9554392; doi:10.1186/s12947-022-00296-z)
Supplement: Supplementary file 1 — Additional file 1. Content of cardiac POCUS lecture. [file 12947_2022_296_MOESM1_ESM.docx]

**Additional File 1** Content of cardiac POCUS lecture

|  | Item | Content | Time |
| --- | --- | --- | --- |
| Part 1 | Basic 5 cardiac POCUS views | Introduction to the 5 cardiac POCUS views (PLAX, PSAX, A4C, S4C, and SIVC) with the ASE online module slides and the 5 POCUS views figures from the WINFOCUS recommendations*. | 2 minutes |
| Part 2 | Acoustic windows | Utilization of 3 acoustic windows (left parasternal, apical, and subcostal windows) to obtain the 5 cardiac POCUS views. | 2 minutes |
| Part 3 | Cardiac ultrasound beam shape | Explanation of triangular-shaped cardiac ultrasound beam. The instructor uses a folding fan and a model heart to illustrate how and why a triangular-shaped beam is emitted from the probe in an easy-to-understand way. | 2 minutes |
| Part 4 | Probe orientation marker | Instruction on how to utilize the probe orientation marker and ultrasound display orientation marker for image acquisition. Students place a HHU probe on a model heart and instructor asks questions about the expected images. | 2 minutes |
| Part 5 | Probe manipulation | Demonstration of 4 probe manipulations (sliding, rotation, angling, and tilting) on a model heart, showing how the ultrasound beam is moved when applying each manipulation using a folding fan as emitted ultrasound beam. The instructor emphasizes the importance of applying just one manipulation at a time to adjust the image. | 2 minutes |
| Part 6 | 5-view anatomy | The question-and-answer review of 40 anatomical structures seen in the 5 views using the pre-training knowledge test questions. The instructor gives feedback for incorrect answers. | 10 minutes |
| Part 7 | 5-view image acquisition instruction | Instructions with a pre-recorded video demonstrating how to obtain the 5 views. In the video, the instructor demonstrates the 5 views by manipulating the probe on his own chest wall and demonstrating correctly obtained views. The instructor provides feedback for students' questions about probe manipulation and image acquisition. The pre-recorded video is in Additional File 2. | 10 minutes |
| *ASE*, American Society of Echocardiography; *A4C*, apical 4-chamber view; *HHU*, handheld ultrasound; *IVC*, inferior vena cava; *PLAX*, parasternal long-axis view; *POCUS*, point-of-care ultrasound, *PSAX*, papillary muscle level of parasternal short-axis view; *SIVC*, subcostal inferior vena cava view; *S4C*, subcostal 4-chamber view; *WINFOCUS*; World Interactive Network Focused on Critical Ultrasound.  Adapted from Jujo et al. *The Pilot and Feasibility Studies.* 2021;7:175. | | | |
