# Supplementary material for: Medical student medium-term skill retention following cardiac point-of-care ultrasound training based on the American Society of Echocardiography curriculum framework
Source: Cardiovasc Ultrasound. 2022 Oct 12;20:26. doi: 10.1186/s12947-022-00296-z (PMC9554392; doi:10.1186/s12947-022-00296-z)
Supplement: Supplementary file 3 — Additional file 3. Main instruction points for 5 cardiac POCUS views image acquisition. [file 12947_2022_296_MOESM3_ESM.docx]

| **Additional File 3** Main instruction points for 5 cardiac POCUS views image acquisition | |
| --- | --- |
| 5 cardiac POCUS views | Probe manipulation instructions |
| PLAX | Place the probe on the 3rd or 4th left intercostal space immediately next to the sternum. And then, rotate the probe so that probe marker points to the patient's right shoulder. |
| PSAX | Start from a clear image of PLAX and rotate the probe 90 degrees clockwise so that the probe marker points to the patient's left shoulder. When rotating from PLAX to PSAX, the fish mouth appearance of the mitral valve level in the short-axis view is usually seen first. And then, tilt the probe downward carefully until both papillary muscles are visualized clearly. |
| A4C | Place the probe on the 5th or 6th left intercostal space under the patient's left nipple. And then, tilt up the probe toward the patient's right shoulder until all 4 chambers and the true longitudinal LV cavity are visualized clearly. (A mild LV foreshortening is acceptable for cardiac POCUS use.) |
| S4C | Place the probe immediately below the xiphoid process and point the probe marker to 3 o'clock direction. And then, tilt up the probe toward the patient's left shoulder until all 4 chambers are visualized clearly. |
| SIVC | Start from a S4C. And then, rotate the probe 90 degrees counterclockwise so that the probe marker points toward the patient's head. |
| *A4C*, apical 4-chamber view; *IVC*, inferior vena cava; *LV*, left ventricle; *PLAX*, parasternal long-axis view; *POCUS*, point-of-care ultrasound, *PSAX*, papillary muscle level of parasternal short-axis view; *SIVC*, subcostal inferior vena cava view; *S4C*, subcostal 4-chamber view. Adapted from Jujo et al. *The Pilot and Feasibility Studies.* 2021;7:175. | |
