# Supplementary material for: Medical student medium-term skill retention following cardiac point-of-care ultrasound training based on the American Society of Echocardiography curriculum framework
Source: Cardiovasc Ultrasound. 2022 Oct 12;20:26. doi: 10.1186/s12947-022-00296-z (PMC9554392; doi:10.1186/s12947-022-00296-z)
Supplement: Supplementary file 8 — Additional file 8. Discriminatory ability of skill test scoring system. [file 12947_2022_296_MOESM8_ESM.pptx]

## Slide 1
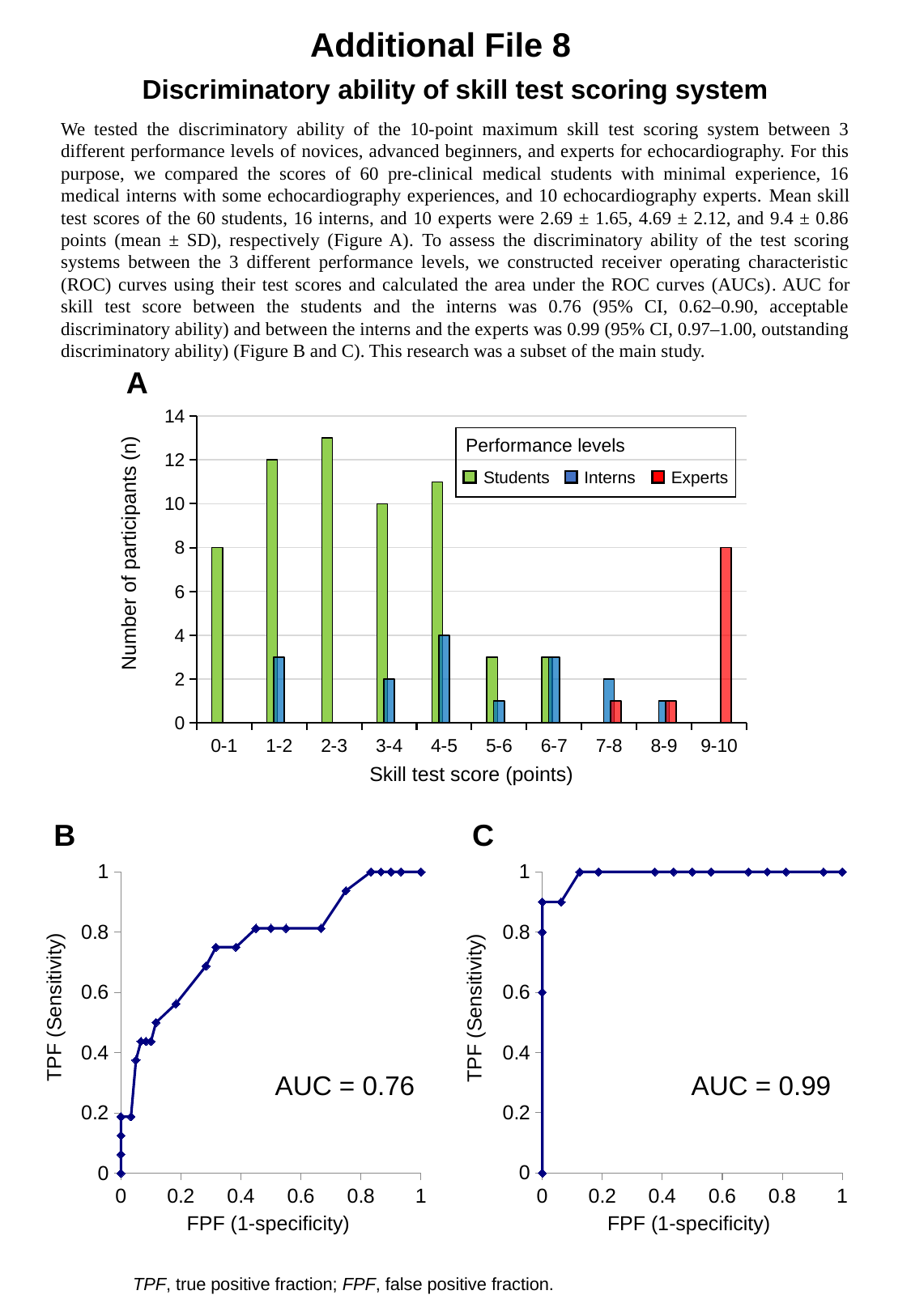

Additional File 8
Discriminatory ability of skill test scoring system
We tested the discriminatory ability of the 10-point maximum skill test scoring system between 3 different performance levels of novices, advanced beginners, and experts for echocardiography. For this purpose, we compared the scores of 60 pre-clinical medical students with minimal experience, 16 medical interns with some echocardiography experiences, and 10 echocardiography experts. Mean skill test scores of the 60 students, 16 interns, and 10 experts were 2.69 ± 1.65, 4.69 ± 2.12, and 9.4 ± 0.86 points (mean ± SD), respectively (Figure A). To assess the discriminatory ability of the test scoring systems between the 3 different performance levels, we constructed receiver operating characteristic (ROC) curves using their test scores and calculated the area under the ROC curves (AUCs). AUC for skill test score between the students and the interns was 0.76 (95% CI, 0.62–0.90, acceptable discriminatory ability) and between the interns and the experts was 0.99 (95% CI, 0.97–1.00, outstanding discriminatory ability) (Figure B and C). This research was a subset of the main study.
A
Number of participants (n)
### Chart
| Category | Student | intern | Expert |
|---|---|---|---|
| 0-1 | 8.0 | None | None |
| 1-2 | 12.0 | 3.0 | None |
| 2-3 | 13.0 | None | None |
| 3-4 | 10.0 | 2.0 | None |
| 4-5 | 11.0 | 4.0 | None |
| 5-6 | 3.0 | 1.0 | None |
| 6-7 | 3.0 | 3.0 | None |
| 7-8 | None | 2.0 | 1.0 |
| 8-9 | None | 1.0 | 1.0 |
| 9-10 | None | None | 8.0 |
Performance levels
Students
Interns
Experts
Skill test score (points)
### Chart
| Category | Skill test score |
|---|---|
### Chart
| Category | Skill test score |
|---|---|B
C
TPF (Sensitivity)
TPF (Sensitivity)
AUC = 0.99
AUC = 0.76
FPF (1-specificity)
FPF (1-specificity)
TPF, true positive fraction; FPF, false positive fraction.
