# Supplementary material for: Medical student medium-term skill retention following cardiac point-of-care ultrasound training based on the American Society of Echocardiography curriculum framework
Source: Cardiovasc Ultrasound. 2022 Oct 12;20:26. doi: 10.1186/s12947-022-00296-z (PMC9554392; doi:10.1186/s12947-022-00296-z)
Supplement: Supplementary file 10 — Additional file 10. Discriminatory ability of knowledge test scoring system. [file 12947_2022_296_MOESM10_ESM.pptx]

## Slide 1
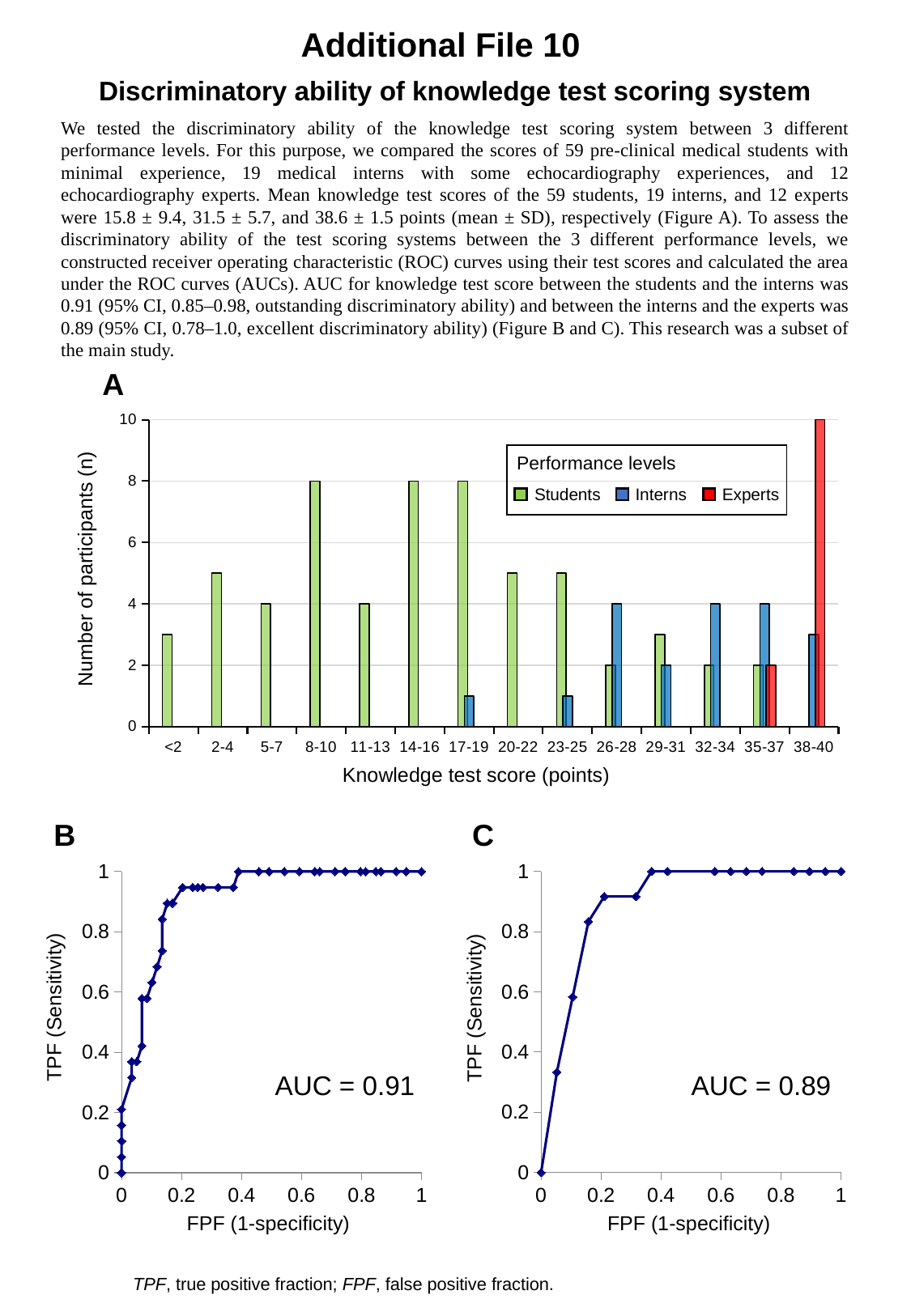

Additional File 10
Discriminatory ability of knowledge test scoring system
We tested the discriminatory ability of the knowledge test scoring system between 3 different performance levels. For this purpose, we compared the scores of 59 pre-clinical medical students with minimal experience, 19 medical interns with some echocardiography experiences, and 12 echocardiography experts. Mean knowledge test scores of the 59 students, 19 interns, and 12 experts were 15.8 ± 9.4, 31.5 ± 5.7, and 38.6 ± 1.5 points (mean ± SD), respectively (Figure A). To assess the discriminatory ability of the test scoring systems between the 3 different performance levels, we constructed receiver operating characteristic (ROC) curves using their test scores and calculated the area under the ROC curves (AUCs). AUC for knowledge test score between the students and the interns was 0.91 (95% CI, 0.85–0.98, outstanding discriminatory ability) and between the interns and the experts was 0.89 (95% CI, 0.78–1.0, excellent discriminatory ability) (Figure B and C). This research was a subset of the main study.
A
Number of participants (n)
### Chart
| Category | Student | Intern | Expert |
|---|---|---|---|
| <2 | 3.0 | None | None |
| 2-4 | 5.0 | None | None |
| 5-7 | 4.0 | None | None |
| 8-10 | 8.0 | None | None |
| 11-13 | 4.0 | None | None |
| 14-16 | 8.0 | None | None |
| 17-19 | 8.0 | 1.0 | None |
| 20-22 | 5.0 | None | None |
| 23-25 | 5.0 | 1.0 | None |
| 26-28 | 2.0 | 4.0 | None |
| 29-31 | 3.0 | 2.0 | None |
| 32-34 | 2.0 | 4.0 | None |
| 35-37 | 2.0 | 4.0 | 2.0 |
| 38-40 | None | 3.0 | 10.0 |
Performance levels
Students
Interns
Experts
Knowledge test score (points)
### Chart
| Category | knowledge score |
|---|---|
### Chart
| Category | knowledge score |
|---|---|B
C
TPF (Sensitivity)
TPF (Sensitivity)
AUC = 0.89
AUC = 0.91
FPF (1-specificity)
FPF (1-specificity)
TPF, true positive fraction; FPF, false positive fraction.
