# Supplementary material for: Medical student medium-term skill retention following cardiac point-of-care ultrasound training based on the American Society of Echocardiography curriculum framework
Source: Cardiovasc Ultrasound. 2022 Oct 12;20:26. doi: 10.1186/s12947-022-00296-z (PMC9554392; doi:10.1186/s12947-022-00296-z)
Supplement: Supplementary file 11 — Additional file 11. GREET checklist. [file 12947_2022_296_MOESM11_ESM.docx]

| **Additional File 11. GREET checklist*** based upon the TIDieR guidance | page |
| --- | --- |
| BRIEF NAME |  |
| 1. INTERVENTION: Provide a brief description of the educational intervention for all groups involved [e.g. control and comparator(s)]. | 1 |
| WHY - this educational process |  |
| 2. THEORY: Describe the educational theory (ies), concept or approach used in the intervention. | 7-8 |
| 3. LEARNING OBJECTIVES: Describe the learning objectives for all groups involved in the educational intervention. | 7-12 |
| 4. EBP CONTENT: List the foundation steps of EBP (ask, acquire, appraise, apply, assess) included in the educational intervention. | 7-12 |
| WHAT |  |
| 5. MATERIALS: Describe the specific educational materials used in the educational intervention.  Include materials provided to the learners and those used in the training of educational intervention providers. | 6-10 |
| 6. EDUCATIONAL STRATEGIES: Describe the teaching / learning strategies (e.g. tutorials, lectures, online modules) used in the educational intervention. | 6-10 |
| 7. INCENTIVES: Describe any incentives or reimbursements provided to the learners. | 7 |
| WHO PROVIDED |  |
| 8. INSTRUCTORS: For each instructor(s) involved in the educational intervention describe their professional discipline, teaching experience / expertise. Include any specific training related to the educational intervention provided for the instructor(s). | 7-8 |
| HOW |  |
| 9. DELIVERY: Describe the modes of delivery (e.g. face-to-face, internet or independent study package) of the educational intervention. Include whether the intervention was provided individually or in a group and the ratio of learners to instructors. | 7-12 |
| WHERE |  |
| 10. ENVIRONMENT: Describe the relevant physical learning spaces (e.g. conference, university lecture theatre, hospital ward, community) where the teaching / learning occurred. | 7 |
| WHEN and HOW MUCH |  |
| 11. SCHEDULE: Describe the scheduling of the educational intervention including the number of sessions, their frequency, timing and duration. | 7-10 |
| 12. Describe the amount of time learners spent in face to face contact with instructors and any designated time spent in self-directed learning activities. | 7-10 |
| PLANNED CHANGES |  |
| 13. Did the educational intervention require specific adaptation for the learners? If yes, please describe the adaptations made for the learner(s) or group(s). | No |
| UNPLANNED CHANGES |  |
| 14. Was the educational intervention modified **during** the course of the study? If yes, describe the changes (what, why, when, and how). | No |
| HOW WELL |  |
| 15. ATTENDANCE: Describe the learner attendance, including how this was assessed and by whom. Describe any strategies that were used to facilitate attendance. | 14-16 |
| 16. Describe any processes used to determine whether the materials (item 5) and the educational strategies (item 6) used in the educational intervention were delivered as originally planned**.** | 14-16 |
| 17. Describe the extent to which the number of sessions, their frequency, timing and duration for the educational intervention was delivered as scheduled (item 11). | 14-16 |

***based on the TIDieR guidance. We strongly recommend reading this statement in conjunction with the GREET 2015 explanation and elaboration paper for important clarifications on all the items. If relevant, we also recommend reading the TIDieR guidance (Hoffman et al. 2014)**
