# Supplementary material for: Medical student medium-term skill retention following cardiac point-of-care ultrasound training based on the American Society of Echocardiography curriculum framework
Source: Cardiovasc Ultrasound. 2022 Oct 12;20:26. doi: 10.1186/s12947-022-00296-z (PMC9554392; doi:10.1186/s12947-022-00296-z)
Supplement: Supplementary file 12 — Additional file 12. Mean skill test scores and breakdown scores in student groups with all tests completed and without 8-week post-training tests completed. [file 12947_2022_296_MOESM12_ESM.docx]

Additional File 12

Mean skill test scores and breakdown scores for 5 cardiac POCUS views in student groups with all tests completed (A) and without 8-week post-training tests completed (B)

A

| Mean skill test scores and breakdown scores in a student group with all tests completed (n = 27) | | | | | |
| --- | --- | --- | --- | --- | --- |
|  | Pre-training | | Immediate post-training | | 8-week post-training |
| Skill test score* | 3.20 ± 1.48 | | 7.85 ± 1.26 | | 4.81 ± 2.28 |
| PLAX score^†^ | 0.75 ± 0.86 | | 1.57 ± 0.58 | | 0.73 ± 0.84 |
| PSAX score^†^ | 0.48 ± 0.70 | | 1.64 ± 0.49 | | 0.52 ± 0.79 |
| A4C score^†^ | 0.32 ± 0.47 | | 1.26 ± 0.41 | | 0.68 ± 0.65 |
| S4C score^†^ | 1.26 ± 0.64 | | 1.42 ± 0.50 | | 1.40 ± 0.61 |
| SIVC score^†^ | 0.38 ± 0.79 | | 1.96 ± 0.14 | | 1.49 ± 0.83 |
|  |  |  | |  | |
| *A4C*, apical 4-chamber view; *PLAX*, parasternal long-axis view; *PSAX*, papillary muscle level of parasternal short-axis view; *SIVC*, subcostal inferior vena cava view; *S4C*, subcostal 4-chamber view.  Data are presented as mean ± SD.  *10-point maximum score, ^†^2-point maximum score.  The 2-point maximum scores for each of the 5 cardiac POCUS views are added for the 10-point maximum skill test score. | | | | | |

B

| Mean skill test scores and breakdown scores in a student group without 8-week post-training tests completed (n = 27) | | | | | |
| --- | --- | --- | --- | --- | --- |
|  | Pre-training | | Immediate post-training | | 8-week post-training |
| Skill test score* | 2.21 ± 1.75 | | 7.96 ± 0.98 | | NA |
| PLAX score^†^ | 0.26 ± 0.60 | | 1.64 ± 0.56 | | NA |
| PSAX score^†^ | 0.49 ± 0.57 | | 1.73 ± 0.26 | | NA |
| A4C score^†^ | 0.32 ± 0.49 | | 0.96 ± 0.53 | | NA |
| S4C score^†^ | 0.86 ± 0.77 | | 1.65 ± 0.42 | | NA |
| SIVC score^†^ | 0.27 ± 0.65 | | 1.98 ± 0.09 | | NA |
|  |  |  | |  | |
| *A4C*, apical 4-chamber view; *NA*, not applicable; *PLAX*, parasternal long-axis view; *PSAX*, papillary muscle level of parasternal short-axis view; *SIVC*, subcostal inferior vena cava view; *S4C*, subcostal 4-chamber view.  Data are presented as mean ± SD.  *10-point maximum score, ^†^2-point maximum score.  The 2-point maximum scores for each of the 5 cardiac POCUS views are added for the 10-point maximum skill test score. | | | | | |
