# Supplementary material for: Medical student medium-term skill retention following cardiac point-of-care ultrasound training based on the American Society of Echocardiography curriculum framework
Source: Cardiovasc Ultrasound. 2022 Oct 12;20:26. doi: 10.1186/s12947-022-00296-z (PMC9554392; doi:10.1186/s12947-022-00296-z)
Supplement: Supplementary file 13 — Additional file 13. Subgroup characteristics of students. [file 12947_2022_296_MOESM13_ESM.docx]

| Additional File 13 Subgroup characteristics of students with a skill test score of 5 points or higher and less than 5 points at 8-week post-training | | | |
| --- | --- | --- | --- |
|  | Students with 5 or higher skill test scores (n = 12) | Students with less than 5 skill test scores (n = 15) | All 27 students (n = 27) |
| 1st year/2nd year student | 9 (75)/3 (25) | 8 (53)/7(47) | 17 (63)/10 (37) |
| Age (years) | 24.8 ± 2.1 | 25.2 ± 4.6 | 25.0 ± 3.6 |
| Female | 2 (17) | 8 (53) | 10 (37) |
| Left hand dominant | 2 (17) | 0 (0) | 2 (7) |
| Pre-training skill test score  (10-point maximum) | 3.31 ± 1.45 | 3.11 ± 1.56 | 3.20 ± 1.48 |
| Pre-training knowledge test score  (40-point maximum) | 19.8 ± 10.0 | 15.0 ± 8.9 | 17.1 ± 9.5 |
| First-choice residency program | GS 4 (33) IM 2 (17) EM 2 (17) Ortho 1 (8) FM 1 (8) Peds 1 (8) IR 1 (8) | IM 4 (27) EM 3 (20) Ortho 3 (20) GS 2 (13) Peds 2 (13) Undecided 1 (7) | IM 6 (22) GS 6 (22) EM 5 (19) Ortho 4 (15) Peds 3 (11) FM 1 (4) IR 1 (4) Undecided 1 (4) |
| Previous ultrasound training experience |  |  |  |
| Structured ultrasound hands-on training or lecture | 0 (0) | 0 (0) | 0 (0) |
| Unstructured ultrasound hands-on training or lecture | 7 (58) | 8 (53) | 15 (56) |
| Cardiac ultrasound on patients | 0 (0) | 1 (7) | 1 (4) |
| Observation of a cardiac ultrasound on patients | 8 (67) | 8 (53) | 16 (59) |
| Cardiac ultrasound on healthy volunteers | 8 (67) | 13 (87) | 21 (78) |
| Cardiac ultrasound on simulators | 1 (8) | 1 (7) | 2 (7) |
| Experience using HHU  (Butterfly iQ) | 1 (8) | 3 (20) | 4 (15) |
| Completion of pre-training self-study of the ASE online module | 12 (100) | 14 (93) | 26 (96) |
| ASE online module review between immediate post-training tests and 8-week post-training tests | 2 (17) | 4 (27) | 6 (22) |
| Review of texbooks or websites other than the ASE module between immediate post-training tests and 8-week post-training tests | 4 (33) | 7 (47) | 11 (41) |
| Additional hands-on training between immediate post-training tests and 8-week post-training tests | 0 (0) | 0 (0) | 0 (0) |
| *ASE*, American Society of Echocardiography; *EM*, emergency medicine; *FM*, family medicine; *GS*, general surgery; *IM*, internal medicine; *IR*, interventional radiology; *Ortho*, orthopedics; *Peds*, pediatrics.  Data are presented as mean ± SD or *n* (%). | | | |
